# How Much Does SARS-CoV-2 Infection during Pregnancy Affect the Neonatal Brain, Heart, and Kidney? A Parallel between COVID-19, Vaccination, and Normal Pregnancy

**DOI:** 10.3390/life14020224

**Published:** 2024-02-05

**Authors:** Daniela Eugenia Popescu, Ana Maria Cristina Jura, Dana Știube, Adrian Ciulpan, Florina Stoica, Simona Ioana Șipoș, Cosmin Cîtu, Florin Gorun, Mărioara Boia

**Affiliations:** 1 Department of Obstetrics and Gynecology, “Victor Babeş” University of Medicine and Pharmacy, Eftimie Murgu Sq. No. 2, 300041 Timisoara, Romania; popescu.daniela@umft.ro (D.E.P.);; 2 Department of Neonatology, Premiere Hospital, Regina Maria Health Network, Calea Aradului, No. 113, 300645 Timisoara, Romania; 3 Department of Pediatrics, Victor Babes University of Medicine and Pharmacy, 300041 Timisoara, Romania; 4 Department of Ophthalmology, Emergency Municipal Clinical Hospital, Gheorghe Dima Street 5, 300254 Timisoara, Romania; florinastoica@gmail.com; 5 Department of Pharmacology, ‘Victor Babes’ University of Medicine and Pharmacy of Timisoara, 300041 Timisoara, Romania

**Keywords:** pregnancy, COVID-19, neonatal, ultrasound, infectious diseases, SARS-CoV-2, vaccine, disease course

## Abstract

During the last decades, a growing number of studies have shown that infections during pregnancy have an important impact on both pregnant women and their fetuses. Our goal was to include newborns from pregnancies with SARS-CoV-2 infection and to investigate the extension of neonatal complications using cardiac, abdominal, and cerebral ultrasonography; hearing testing; and indirect ophthalmoscopy. Likewise, neonates whose mothers were vaccinated against COVID-19 during pregnancy and those from pathology-free pregnancies were examined. A total of 458 mother–newborn dyads were included over a period of 10 months and divided into three groups: the COVID-19 group, vaccine group, and control group. Although six cardiac malformations were found in the COVID-19 group, no correlation was made compared to the vaccine and control group (*p* = 0.07). Grade 1 intraventricular hemorrhage and hypoxic ischemic encephalopathy were the most prevalent among neonates from mothers with SARS-CoV-2 infection (*p* = 0.002 and *p* < 0.001, respectively). The kidney anomaly found to be most frequent in this group was grade 1 unilateral hydronephrosis (*p* < 0.001). COVID-19 disease during the gestational period had no effect on the auditory or visual function. Our findings highlight the importance of implementing proper infection control practices for future mothers, and by continuing to investigate this topic, we can gather valuable insights that will improve neonatal health in this context.

## 1. Introduction

During the last decades, a growing number of studies have shown that infections acquired during pregnancy have an important impact on both pregnant women and their fetuses, with increased risk of complications in neonatal outcomes [1]. The first case of human coronavirus disease (COVID-19) in humans, which was caused by SARS-CoV-2 (severe acute respiratory syndrome coronavirus), was reported in December 2019 [2]. Since then, SARS-CoV-2 has affected over 765 million people worldwide, causing more than 6.9 million deaths. Among the pregnant population, its prevalence has been reported to be approximately 14% to 15%, with over 220,000 cases, most of them being asymptomatic [3].

In utero SARS-CoV-2 transmission has been the topic of discussion since the start of the global health crisis. Third-trimester fetal infection was reported to occur in 3.2% of cases [4]. The primary action mechanism that leads to vertical transmission involves angiotensin-converting enzyme 2 (ACE2) and S protein protease receptors, which facilitate virus entry into the cell. These receptors can be found in developing human embryos during the early stages of development. As a result, SARS-CoV-2 has the ability to penetrate fetal cells in the preliminary phases of evolution and affect cell transformation and growth, leading to early intrauterine infection [5]. 

COVID-19 vaccination during pregnancy has been carefully managed due to the fact that pregnant women were initially excluded from the vaccine trials. To date, vaccination against SARS-CoV-2 infection plays a crucial role in preventing maternal illness. While several observational studies examined neonatal benefits via antibodies transfer across the placenta, large-scale assessments of neonatal safety are scarce [6].

Viruses such as varicella and rubella can cross the placenta and harm the fetus. Miscarriage, growth restriction, hydrops, and even death are all possible outcomes of fetal viral infection. During the first 6 gestational weeks, SARS-CoV-2 confers a higher chance of developing congenital birth defects [5]. Compared to the evidence that influenza infection during the first trimester of pregnancy is teratogenic (more precisely hyperthermia and fever), it is presumed that fever associated with COVID-19 in the first trimester could induce congenital anomalies. Limited data about the effects of severe SARS-CoV-2 infection during the first trimester of pregnancy on the risk of major congenital malformations are available [7,8]. 

Therefore, our goal for this study was to identify newborns from pregnancies with SARS-CoV-2 infection and to investigate the extension of neonatal complications using cardiac, abdominal, and cerebral ultrasonography; hearing testing; and indirect ophthalmoscopy. Similarly, neonates whose mothers were vaccinated against COVID-19 during pregnancy and those from pathology-free pregnancies were examined. 

## 2. Materials and Methods

Our study was conducted over a period of 10 months (November 2021–August 2022) in the Clinic of Neonatology of the Première Hospital, Regina Maria Health Network, Timișoara, Romania, where both birth and neonatal follow-up took place. This study was approved by the Première Hospital’s Ethics Commission Board (No. 330/18.11.2021), Timișoara, Romania, as well as by the Ethics Committee of Scientific Research of “Victor Babeș” University of Medicine and Pharmacy Timișoara (No. 76/2020), Romania. The patients agreed and signed an informed consent form that followed the guidelines of the Declaration of Helsinki. This study was approved by our institutional review board. 

A total of 458 newborns were included, divided into three groups: The COVID-19 group included 167 newborns from mothers with SARS-CoV-2 infection during pregnancy; the vaccination group consisted of 91 neonates born from mothers immunized against SARS-CoV-2 during the gestational period; and the control group included 200 newborns. 

During the study period, two SARS-CoV-2 variants of concern (VOCs) were present in Romania (Omicron B.1.1.529 and Delta AY.4.2), but in our country, phenotype testing is not mandatory; therefore, only RT-PCR testing was performed.

Inclusion criteria were represented by maternal COVID-19 infection during pregnancy, confirmed using RT-PCR testing for the first group, immunization against SARS-CoV-2 with BioNTech-Pfizer or Moderna vaccine during the gestational period for the second group, and no acquired infections or vaccination during pregnancy for the third group, respectively. Exclusion criteria were represented by vaccination during pregnancy along with SARS-CoV-2 infection or any other infection acquired during the gestational period, neonates born to emergency deliveries, and perinatal complications such as prolonged labor or asphyxia. No twins or multiple-gestation pregnancies were included. The investigations were conducted during the first four days of life.

The study aimed to evaluate the changes that occurred after maternal infection and vaccination during pregnancy, mainly regarding the neonatal heart, brain, and kidney using ultrasonography; hearing impairment using evoked otoacoustic emissions along with auditory brainstem response (EOAE-ABR) screening; and ocular anomalies using indirect ophthalmoscopy compared to the control group. 

The hearing screening test results were quantified as “PASS” for those who passed the test and “REFER” for those who did not. All patients underwent eye examination and were investigated by our ophthalmologist specialized in neonatal indirect ophthalmoscopy.

For the cerebral and ultrasonographic examinations, Voluson E8 (GE Healthcare, Austria GmbH&Co OG, Chicago, IL, USA) equipment with microconvex transducer at a bandwidth of 3–9 MHz was used. The investigations were performed by certified ultrasonographists: a neonatologist with ultrasonography certification for the cerebral and abdominal examination and a pediatric cardiologist for echocardiographic exams. Cerebral ultrasonography evaluated brain structures, hemodynamic parameters of cerebral arteries, dimensions of ventricles, and pericerebral fluid spaces. In relation to the identified cases of hydronephrosis, the severity was assessed according to the Society of Fetal Urology’s numerical grading system. 

Transthoracic cardiac ultrasound was performed using a Samsung HS 60 ultrasound scanne (Samsung Medison Co., Seoul, Republic of Korea), and a PA3-8B phased array probe, with a frequency between 3 and 8 MHz.

Follow-up tests were carried out during 21 and 28 days of life, as our hospital offers a free, although not mandatory, follow-up up to 28 days of life to newborns born in our unit. Therefore, only a small percent returned for reevaluation (38%). Also, a lot of our patients chose to give birth in our clinic, although their residence is not in the same city as our hospital.

### Statistical Analysis

Categorical data are summarized as counts and percentages. An a priori power analysis was conducted with the objective of ensuring sufficient power to detect meaningful differences within and between groups. Using R studio software, version 2023.09.1+494 (2023.09.1+494) for Mac, with package “pwr”, we calculated the required sample size based on a medium effect size, as suggested by Cohen’s guidelines, and the standard deviations obtained from preliminary data. This sample size calculation is crucial for ensuring that the study is adequately powered to detect a clinically significant difference between groups if one exists [9,10]. The power of 80% was targeted to minimize the risk of Type II errors, meaning that we aimed to reduce the likelihood of falsely accepting the null hypothesis when in fact a true difference exists. The 5% significance level was chosen to control the risk of Type I errors, i.e., falsely rejecting the null hypothesis when it is true. The analysis indicated that to achieve 80% power at a 5% significance level and a Cohen’s effect size of 0.5 for our primary outcome, a sample size of 63.76 (64) participants per group was indicated. Even though the power analysis suggested a minimum of 64 participants per group to achieve the desired power, our study included more participants to ensure a higher statistical power. This increases the likelihood of detecting a true effect if present and reduces the risk of Type II errors. 

The regression coefficient (R), R^2^ (coefficient of determination), and *p*-values for individual predictors were calculated assess the strength of association between variables. A *p*-value less than 0.05 was considered statistically significant. Differences between groups for continuously normal data were tested using Welch’s *t*-test for two groups or ANOVA if there were more than two groups. Differences between categorical data were tested using the test or Fisher’s exact test when the expected cell count was less than five. We performed a binomial logistic regression to determine independent risk factors. The selection of cases was guided by the lowest Akaike information criterion (AIC), which helps in identifying models that best explain the data with minimal information loss. A backward regression model was used, which involves starting with all potential predictors and systematically removing those that contribute the least to the model’s predictive power. This approach ensures a more efficient and accurate model by retaining only the most significant variables. All statistical analysis was performed with R (version 3.6.3) using the “gtsummary” and “V8” packages. 

## 3. Results

Our research compared the effects of SARS-CoV-2 infection during pregnancy with COVID-19 vaccination and normal pregnancies on the neonatal brain, heart, and kidney using ultrasonographic examinations during the first 4 days after birth, together with auditory screening and ophthalmologic evaluation.

Neonates born to emergency deliveries and with perinatal complications such as prolonged labor or asphyxia were excluded from the study. No twins or multiple-gestation pregnancies were included. 

The mean maternal age was 32.56 years (control group = 33.06 years, COVID-19 group = 32.49 years, and vaccine group = 32.13 years). Most of the vaccination took place during the third trimester of pregnancy (*n* = 42), followed by the second trimester (*n* = 27) and the first (*n* = 22). As for the infection period, a large percent of cases contracted the disease during the third and second trimesters (68 vs. 66 cases), followed by the first trimester (33 cases). Most of them had mild symptoms (*n* = 120), 42 experienced moderate symptoms including fever, and 2.99% (*n* = 5) required oxygen therapy and admission. All mothers received vitamin therapy during the infection period (100%), 25.1% received antipyretics, and only a few (2.99%) received anticoagulant and antiviral medication (nirmatrelvir/ritonavir). The most prevalent signs of disease were represented by flu-like symptoms such as fatigue, chills, pharyngitis, headaches, rhinorrhea, myalgia, and anosmia. 

**Figure 1:**
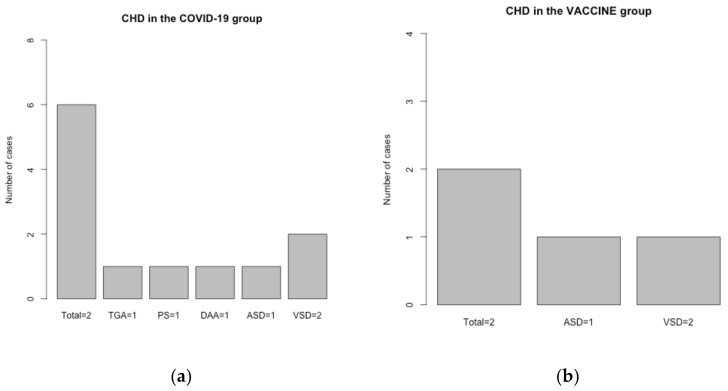
(**a**) Congenital heart disease (CHD) prevalence in the COVID-19 group. TGA, transposition of the great arteries; PS, pulmonary stenosis; DAA, double aortic arch; ASD, atrial septal defect; VSD, ventricular septal defect. (**b**) Congenital heart disease (CHD) prevalence in the vaccine group. ASD, atrial septal defect; VSD, ventricular septal defect.

The cardiac ultrasonography was performed by a pediatric cardiologist in all cases between the first and the fourth day after birth, respectively. A great number of newborns, i.e., 152 (91%), from the COVID-19 group had normal echocardiographic exams compared to the vaccine group with 86 (95%) normal evaluations and the control group with 190 (95%). The pathological findings were separated into two categories: patent foramen ovale (PFO) and congenital heart disease (CHD). For the persistent foramen ovale class, eight cases were found in the COVID-19 group (4.8%), three cases in the vaccine group (3.3%), and nine in the control group (4.5%). No association was achieved for this category (*p* > 0.9). As for congenital heart malformations, only one occurred in the control group (0.5%), two in the vaccine group (2.2%), and six in the COVID-19 group (3.6%). Although the number of CHD is greater than other groups, no statistical difference was found (*p* = 0.07), as seen in Table 1. A detailed graphic of the CHDs found in the COVID-19 group can be observed in Figure 1a, and the main CHDs found in the vaccine group can be observed in Figure 1b. It is necessary here to specify that for transposition of the great arteries, pulmonary stenosis, and double aortic arch, the SARS-CoV-2 infection occurred during the first trimester of pregnancy, while for atrial septal defect and ventricular septal defect cases, the infection was acquired during the second trimester. Three patients from the CHD group came from in vitro fertilization pregnancies.

The incidence of CHDs in Romania is generally around 0.7%, and our unit reports 0.5%, which is below the national average.

Cerebral ultrasonography detected several anomalies, from intraventricular hemorrhage (IVH) (grade 1, 2, and 3) to hypoxic ischemic encephalopathy (HIE) (grade 1, 2, and 3), choroid plexus cysts, severe ventriculomegaly, and cerebral infarction. The most prevalent abnormality was represented by grade 1 intraventricular hemorrhage and grade 1 hypoxic ischemic encephalopathy for all groups. 

Intraventricular hemorrhage grade 1 appeared as the most frequent type of lesion, with 24 cases (14%) in the COVID-19 group, 12 cases in the control group (6%), and only 3 in the vaccine group (3.3%), making a difference among ultrasonographic changes for those born from SARS-CoV-2 infection during pregnancy (*p* < 0.002). Grade 2 and grade 3 IVH were not that common, with three findings among the COVID-19 group (1.8%) for grade 2 and one (0.6%) for grade 3 and almost none for the other groups. 

For the hypoxic ischemic encephalopathy injury, the differences were with greater disparities. We determined statistically significant data for grade 1 HIE patients: 35 within the COVID-19 group (21%) versus 12 in the control group (6%) and 2 in the vaccine group (2.2%), resulting in a *p*-value of <0.001 and demonstrating how the infection during the gestational period can induce bleeding disorders (Table 2). 

Hypoxic ischemic encephalopathy grade 2 and 3 between groups did not have such a great difference, with fewer cases and no statistical relevance. 

Additionally, we grouped together the HIE subtype cases and compared the results across the studied categories (control vs. COVID-19 vs. vaccine as 18 vs. 49 vs. 6), which led to interesting results that suggest an important difference (*p* < 0.001). The same was conducted for the IVH subtype cases (13 vs. 28 vs. 3), suggesting similar results (*p* < 0.001).

Other cerebral abnormalities found consisted of choroid plexus cyst, with six cases in the COVID-19 group (3.6%), which was more than the vaccine group (1.1%) or the control group (0.5%). Severe ventriculomegaly was observed in two cases only: one of the COVID-19 group patients (0.6%) and one of the vaccine group (1.1%). Cerebral infarction was solely seen in a COVID-19-group patient (0.6%). 

Although the main cerebral findings described in our study comprise intraventricular hemorrhage and hypoxic ischemic encephalopathy, we strongly feel the need to mention that all pregnancies were closely monitored by experienced obstetricians, leading to the exclusion of neonates born with perinatal complications such as perinatal asphyxia, prolonged labor, or any other noticeable drawbacks.

In Table 2, COVID-19 group comprised the most cerebral ultrasonographic anomalies, with only 98 normal examinations (59%) compared to the vaccine group (88%) or the control group (85%), suggestive of a greater risk of unfavorable neonatal outcome in cases with SARS-CoV-2 infection during pregnancy (*p* < 0.001). 

Abdominal ultrasonography observed a number of anomalies, such as unilateral and bilateral hydronephrosis, kidney duplication, megaureter, pyeloureteral duplication, horseshoe kidney, and septate gallbladder.

The most common finding among the COVID-19 group of patients was grade 1 unilateral hydronephrosis with 27 cases (16%), while the vaccine group had only three cases (3.3%), and the control group had, 11 cases, i.e., almost one-third (5.5%), suggesting a possible connection between SARS-CoV-2 infection during pregnancy and the fetal and neonatal kidney (*p* < 0.001), as shown in Table 3. 

Grade 2 and 3 unilateral hydronephrosis were seen in much smaller numbers in all groups, accounting for one case in the COVID-19 group (0.6%) for grade 2 and four cases (2.4%) for grade 3, two cases for the vaccine group (2.2%) and two for the control group (1%) for grade 2, and no cases for unilateral grade 3 hydronephrosis. When all subtype cases of hydronephrosis (uni- and bilateral) were brought together and compared across the studied groups (control vs. COVID-19 vs. vaccine as 16 vs. 38 vs. 5), this led to a greater impact across groups (*p* < 0.001).

Bilateral hydronephrosis, other kidney anomalies, and septate gallbladder were seen in small numbers for each group, but altogether, the COVID-19 group had the higher incidence for all of the above. As a consequence, only 124 patients (74%) of the COVID-19 group had normal abdominal ultrasonographic exams compared to the vaccine group with 82 (90%) and the control group with 183 normal investigations (92%), suggesting a possible correlation between maternal SARS-CoV-2 infection during the gestational period and the neonatal abnormalities identified (*p* < 0.001). 

A backward binomial logistic regression model was performed to assess whether gestational age, APGAR score, birthweight, and maternal age could have an impact on ultrasonographic findings. No statistical significance was found (OR 1, 95% CI [0.99, 1.12], *p*-value: 0.2118). 

For our study population, all patients were tested for hearing impairment using otoacoustic emissions along with auditory brainstem response (EOAE-ABR) screening. None of the COVID-19 group and vaccine group presented a negative test result. Only four patients from the control group had a refer result (meaning that the newborn did not pass the hearing screening test) after the first examination and a positive test during the repeated examination after several weeks of life, indicating no effect of SARS-CoV-2 infection during pregnancy or vaccination on the ability to hear.

The study found no ocular anomalies in neonates from SARS-CoV-2-infected mothers during pregnancy compared to the other assessed groups, suggesting infection during the gestational period has no effect on eye abnormalities. 

## 4. Discussion

One of the first hypotheses tested during this study was how COVID-19 disease during pregnancy can impact the fetal heart. Due to the fact that SARS-CoV-2 is considered to have a teratogenic effect if acquired during the first trimester of pregnancy, presumably as a consequence of fever, congenital anomalies were investigated [7]. Goncu et al. showed that the fetal heart does not seem to be negatively affected by moderate COVID-19 after recovery [11]. Our research shows that even though six congenital heart diseases (CHDs) occurred in the COVID-19 group, namely two in the vaccine group and one in the control group, the normal ultrasonographic examination revealed no significant difference between groups (*p* = 0.3). Goldshtein et al. investigated whether the vaccine against SARS-CoV-2 could be associated with congenital heart disease compared to the control group. Consequently, there was no statistically significant difference in the risk of any congenital malformations (RR = 0.69, 95% CI [0.44–1.04]) or heart malformations (RR = 0.75, 95% CI [0.43–1.26]). The exposed group had a lower risk of major cardiac malformations (RR = 0.46, 95% CI [0.24–0.82]) [6]. The study conducted by Murat et al. found no evidence of teratogenic effects on the nervous system or heart caused by COVID-19 infection during pregnancy [12].

Seven newborns with complicated congenital heart and lung malformations born to women who had positive tests for SARS-CoV-2 were described by Goldshtrom et al. Interestingly, out of the seven, three women denied having ever experienced COVID-19 symptoms and were asymptomatic throughout their hospital stay [13].

Three of the nine congenital heart disease patients came from in vitro fertilization pregnancies, indicating a strong possibility for assisted reproductive technologies to be the main reason for this outcome, as suggested by the literature [14].

Patent foramen ovale was seen in all three groups, but from our perspective, the reason for its manifestation can mainly be attributed to the examination being performed within the first 24 to 96 h of life, although Douarte et al. similarly observed that the following results were obtained from a heart ultrasonographic exam that was carried out on 36 newborns: ventricular septal defect (2.7%), atrial septal defect (27.7%), and patent foramen ovale (69.4%) [15]. The ACE2 receptors, which serve as a gate for SARS-CoV-2 to enter cells, are not expressed in fetal heart tissue, according to a study that investigated fetal tissues and their ACE2 receptors. Thus, it appears that the fetal heart is not the direct target of SARS-CoV-2 [16]. Another study revealed that structural defects such as ventricular septal defect and arch hypoplasia do not appear to be connected to COVID-19. Additionally, none of the infants born to mothers infected with SARS-CoV-2 experienced cardiac dysfunction such as myocarditis or cardiomyopathy [17]. 

According to the literature, SARS-CoV-2 infection revealed no association between different prenatal brain growth patterns and cortical development during pregnancy. This observation was made for women who had mild symptoms [18]. Likewise, one study revealed that even though perinatal HIE was more common in the group with severe COVID-19 course, there were no differences between the group with mild disease and the control group in terms of perinatal outcomes [19]. In our study, HIE grade 1 was the most frequent occurrence in the COVID-19 group versus the vaccine and control groups. One study conducted by Norman M. et al. in Sweden showed that maternal SARS-CoV-2 infection in pregnancy was significantly associated with small increases in some neonatal morbidities, such as hyperbilirubinemia, assisted ventilation, and persistent pulmonary hypertension [20].

We identified one case of severe ventriculomegaly in the COVID-19 group and one in the vaccine group. Archuleta et al. described two cases of severe ventriculomegaly, neurological dysfunction, and seizures, which were found in neonates with prenatal exposure to COVID-19 infection during the first and third trimesters of pregnancy [21]. Three fetal anomalies—ventriculomegaly, hydronephrosis, and spina bifida—were reported by Blakeway et al. in women who received the COVID-19 vaccination. The spina bifida case was identified prior to vaccination. Fetal cerebral MRI confirmed that the case of ventriculomegaly, which was diagnosed at 37 weeks of gestation, was isolated and did not have any associated cerebral anomalies. The hydronephrosis was mild, and there were no associated abnormalities [22]. 

In a paper by Kurokawa et al., 90 neonates (with two pairs of twins) and their mothers with SARS-CoV-2 infection were studied. The neonates were divided into symptomatic and asymptomatic groups. The most common ultrasonographic finding was intracranial hemorrhage, more precisely germinal matrix hemorrhage (22.2%), parenchymal hemorrhage (22.2%), and intraventricular hemorrhage (11.1%), along with HIE [23]. Duarte et al. described the following outcomes in 35 newborns undergoing cerebral ultrasonography: no abnormal findings (71.0%), ventricular dilatation (2.8%), grade I or II periventricular hemorrhage (22.0%), and linear calcifications (2.8%) [15]. Our findings revealed a high prevalence of grade 1 intraventricular hemorrhage in the COVID-19 group compared to the vaccine and control groups. 

A small case report carried out by Benny M. et al. described two newborns with early-onset seizures (first day of life), acquired microcephaly, and a notable developmental delay over time. They were born to mothers who tested positive for SARS-CoV-2. Cystic encephalomalacia and severe parenchymal atrophy were revealed by sequential MRI [24].

A possible consequence of severe SARS-CoV-2 infection is the emergence of a worsened thrombophilic state, and cerebral venous thrombosis (CVT) is an uncommon but potential side effect of SARS-CoV-2 infection. This has been observed in both adults and children. Campi et al. described a case study detailing the clinical development of a term newborn with extended CVT of unknown origin, whose mother had contracted SARS-CoV-2 during the third trimester of pregnancy [25]. Our study found one (0.6%) case of cerebral infarction and six (3.6%) cases of choroid plexus cysts, probably suggesting intrauterine coagulation disorders. 

Another paper included 201 newborns from SARS-CoV-2-infected mothers during the gestational period and compared them to 18 unexposed controls. The researchers conducted neurological imagistic examinations at 6 months of adjusted chronological age and revealed 18 grayscale and 21 Doppler abnormalities. The most important observations were hyperechogenicity of deep-brain white matter and basal ganglia and a reduction in the resistance and pulsatility indices of intracranial arterial flow [26].

COVID-19 is mainly the source of respiratory disease, but it was observed that the disease can also lead to thrombogenic ischemia in different organs, including the gastrointestinal tract [27].

In our study, the most important renal anomaly found in the COVID-19 group was unilateral grade 1 hydronephrosis, with 27 (16%) cases compared to the vaccine or control groups. To our knowledge, no studies correlating SARS-CoV-2 intrauterine infection and neonatal hydronephrosis have been published so far. With regard to this topic, in a paper published by He et al., increased levels of both cystatin C and β2-microglobulin were observed in studied newborns, indicating that COVID-19 infection during pregnancy may lead to fetal kidney damage [28]

One study assessing hearing screening test for 65 infants born to mothers infected with COVID-19 during pregnancy revealed that 11 failed the standard hearing screening test, so a second test was performed. Only two neonates required further investigation after the second test [12]. Similarly, our paper shows no effect of SARS-CoV-2 infection during pregnancy on hearing impairment. 

Morhart et al. described 27 newborns of pregnancies with acquired SARS-CoV-2 infection. One mother delivered a newborn with a severe eye malformation, later diagnosed with microphthalmia, microcornea, and hypoplasia of both the optic nerve and the neurosensory retina. Similar unilateral or bilateral eye abnormalities can be observed in neonates with rubella embryopathy [29]. In our study, no eye anomalies were detected from the COVID-19 group or the other comparative groups. 

Our study’s limitations refer to time constraints due to the fact that not all patients came back for a second ultrasonographic evaluation, so no conclusion could be drawn regarding the long-term effects of SARS-CoV-2 or vaccination, respectively. Furthermore, no antibodies against COVID-19 were assessed for the neonates of the control group, suggesting that we cannot exclude a possible asymptomatic infection for those mothers. 

## 5. Conclusions

Our study aimed to compare the effects of SARS-CoV-2 infection during pregnancy with COVID-19 vaccination and normal pregnancies on the neonatal brain, heart, and kidney using ultrasonographic examinations during the first 4 days after birth, together with auditory screening and ophthalmologic evaluation. The main findings were that the fetal heart does not seem to be negatively affected, although six cardiac malformations were found in the COVID-19 group, and no correlation was made compared to the vaccine and control groups. Grade 1 intraventricular hemorrhage and hypoxic ischemic encephalopathy were the most prevalent among neonates from mothers with SARS-CoV-2 infection. The kidney anomaly found to be most frequent in this group was grade 1 unilateral hydronephrosis. COVID-19 disease during the gestational period had no effect on the auditory or visual function. Time constraints are one of our study’s limitations mainly because not all patients returned for a second ultrasonographic evaluation, making it impossible to draw conclusions about the long-term effects of either the vaccination or SARS-CoV-2 infection during pregnancy. Despite the fact that our observations are relevant, further research is needed to fully understand the long-term effects of SARS-CoV-2 infection on pregnancy outcomes and infant development. Our findings highlight the importance of implementing proper infection control practices for future mothers, and by continuing to investigate this topic, we can gather valuable insights that will improve neonatal health in this context.

## Figures and Tables

**Table 1:** Echocardiographic evaluation comparison between groups.

Characteristic	Control Group, *N* = 200 ^1^	COVID-19 Group, *N* = 167 ^1^	Vaccine Group, *N* = 91 ^1^	*p*-Value ^2^
Normal echocardiography	190 (95%)	152 (91%)	86 (95%)	0.3
PFO (Persistent foramen ovale)	9 (4.5%)	8 (4.8%)	3 (3.3%)	>0.9
CHD (Congenital heart disease)	1 (0.5%)	6 (3.6%)	2 (2.2%)	0.07

^1^ *n* (%). ^2^ Pearson’s chi-square test; Fisher’s exact test.

**Table 2:** Cerebral ultrasonographic comparison between groups.

Characteristic	Control Group, *N* = 200 ^1^	COVID-19 Group, *N* = 167 ^1^	Vaccine Group, *N* = 91 ^1^	*p*-Value ^2^
Normal cerebral ultrasonography	170 (85%)	98 (59%)	80 (88%)	<0.001
Intraventricular hemorrhage grade 1	12 (6%)	24 (14%)	3 (3.3%)	0.002
Intraventricular hemorrhage grade 2	1 (0.5%)	3 (1.8%)	0 (0%)	0.4
Intraventricular hemorrhage grade 3	0 (0%)	1 (0.6%)	0 (0%)	0.6
Hypoxic ischemic encephalopathy grade 1	12 (6%)	35 (21%)	2 (2.2%)	<0.001
Hypoxic ischemic encephalopathy grade 2	6 (3%)	12 (7.2%)	3 (3.3%)	0.2
Hypoxic ischemic encephalopathy grade 3	0 (0%)	2 (1.2%)	1 (1.1%)	0.2
Choroid plexus cyst	1 (0.5%)	6 (3.6%)	1 (1.1%)	0.073
Severe ventriculomegaly	0 (0%)	1 (0.6%)	1 (1.1%)	0.3
Cerebral infarction	0 (0%)	1 (0.6%)	0 (0%)	0.6

^1^ *n* (%). ^2^ Pearson’s chi-square test; Fisher’s exact test.

**Table 3:** Abdominal ultrasonographic comparison between groups.

Characteristic	Control Group, *N* = 200 ^1^	COVID-19 Group, *N* = 167 ^1^	Vaccine Group, *N* = 91 ^1^	*p*-Value ^2^
Normal abdominal ultrasound	183 (92%)	124 (74%)	82 (90%)	<0.001
Unilateral grade 1 hydronephrosis	11 (5.5%)	27 (16%)	3 (3.3%)	<0.001
Unilateral grade 2 hydronephrosis	2 (1.0%)	1 (0.6%)	2 (2.2%)	0.4
Unilateral grade 3 hydronephrosis	0 (0%)	4 (2.4%)	0 (0%)	0.043
Bilateral grade 1 hydronephrosis	3 (1.5%)	5 (3.0%)	0 (0%)	0.3
Bilateral grade 2 hydronephrosis	0 (0%)	1 (0.6%)	0 (0%)	0.6
Kidney duplication	3 (1.5%)	3 (1.8%)	1 (1.1%)	>0.9
Megaureter	0 (0%)	1 (0.6%)	0 (0%)	0.6
Pyeloureteral duplication	0 (0%)	1 (0.6%)	0 (0%)	0.6
Septate gallbladder	0 (0%)	1 (0.6%)	1 (1.1%)	0.3
Horseshoe kidney	0 (0%)	1 (0.6%)	1 (1.1%)	0.3

^1^ *n* (%). ^2^ Pearson’s chi-square test; Fisher’s exact test.

## Data Availability

Data are contained within the article.
